# Association Between Psychological Factors and Masticatory Muscle Thickness Assessed by Ultrasonography in Female Dental Students with TMD-Related Myalgia: A Cross-Sectional Study

**DOI:** 10.3390/jcm15093183

**Published:** 2026-04-22

**Authors:** Marcin Wójcicki, Monika Litko-Rola, Jacek Szkutnik, Michał Ginszt, Piotr Gawda, Ingrid Różyło-Kalinowska

**Affiliations:** 1 Independent Unit of Functional Masticatory Disorders, Medical University of Lublin, 20-093 Lublin, Poland; monika.litko-rola@umlub.edu.pl (M.L.-R.); jacek.szkutnik@umlub.edu.pl (J.S.); 2 Department of Stomatognathic System Rehabilitation, Medical University of Lublin, 20-093 Lublin, Poland; michal.ginszt@umlub.edu.pl; 3 Department of Sports Medicine, Medical University of Lublin, 20-093 Lublin, Poland; piotr.gawda@umlub.edu.pl; 4 Department of Dental and Maxillofacial Radiodiagnostics with Digital Dentistry Lab., Medical University of Lublin, 20-093 Lublin, Poland; ingrid.rozylo-kalinowska@umlub.edu.pl

**Keywords:** temporomandibular disorders, myalgia, masticatory muscles, depression, anxiety, ultrasonography

## Abstract

**Objectives:** The aim of this study was to compare the relationship between psychological factors and the thickness of masticatory muscles in patients with temporomandibular disorders (TMD) and healthy controls. **Methods**: This cross-sectional study included 106 female participants aged 18–35 years, recruited from students of the Medical University of Lublin. The study group consisted of individuals diagnosed with myalgia based on the Diagnostic Criteria for Temporomandibular Disorders (DC/TMD), while the control group included participants without TMD. Psychological factors were assessed using the PHQ-9, GAD-7, and PHQ-15 questionnaires. The thickness of the temporalis and masseter muscles was measured using an M-Turbo (Sonosite) ultrasound device under relaxed and contracted conditions. **Results**: Participants with TMD-related myalgia scored significantly higher on the PHQ-9 (mean ± SD: 8.82 ± 5.02 vs. 5.00 ± 3.41; *p* < 0.001), GAD-7 (8.53 ± 4.48 vs. 4.65 ± 3.58; *p* < 0.001), and PHQ-15 (9.47 ± 4.07 vs. 6.02 ± 3.89; *p* < 0.001) compared to controls. No statistically significant differences were observed in temporalis or masseter muscle thickness at rest or during contraction between groups (*p* > 0.05). Significant differences in correlations were found between depression scores and temporalis muscle thickness in the study group compared to the control group (r = 0.32 vs. −0.13, *p* = 0.011) as well as between anxiety scores and temporalis muscle thickness (r = 0.25 vs. −0.30, *p* = 0.002) No significant associations were found for the masseter muscle. **Conclusions**: Individuals with myalgia did not show increased muscle thickness compared to healthy individuals. Psychological distress, particularly depression and anxiety, was differentially related to temporalis muscle morphology in individuals with myalgia compared to healthy controls.

## 1. Introduction

Temporomandibular disorders (TMD) according to the American Dental Association is a collective term used to describe functional disturbances of the masticatory system, including the temporomandibular joints, masticatory muscles, and associated structures [1]. These disorders are characterized by a similar location of symptoms and shared clinical manifestations. The most common symptoms include pain in the head and facial region and limitations in jaw mobility, while joint sounds (e.g., clicking or crepitation) may also occur in some patients [2]. TMD affects 34% of the general population, and projections suggest an increase to 44% by 2050 [3]. The most frequently occurring subgroup within TMD, accounting for 45.3% consists of masticatory muscle pain, also known as myofascial pain [2].

The etiology of TMD is currently understood within the biopsychosocial model, which assumes that biological, psychological, and social factors interact in the development and persistence of the disorder. Biological factors include anatomical characteristics, muscle activity, occlusal conditions, and functional loading of the masticatory system. Demographic factors such as age and sex have also been associated with the prevalence of TMD, with the condition occurring more frequently in young and middle-aged adults and significantly more often in women. Psychological factors such as stress, depression, anxiety, and somatization are also considered important contributors to the onset and maintenance of TMD symptoms [1].

Muscular TMD is often associated with masticatory muscle hypertrophy. Muscle growth is stimulated by mechanical tension, cell damage, or metabolic stress, usually from excessive muscle work, hypoxia, or lactic acid buildup. In masticatory muscles, parafunctional activity is the main trigger [4]. Muscle mass increases through hyperplasia (fiber multiplication) and hypertrophy (fiber enlargement), with the latter being dominant. Hypertrophy occurs as an adaptation to overload, involving increased contractile proteins (actin, myosin) and possibly sarcoplasmic hypertrophy, where muscle volume grows without a proportional gain in strength [4].

From the perspective of human anatomy and physiology, mental state plays a significant role in the development and progression of TMD [5,6]. In recent years, numerous studies have demonstrated a relationship between various psychological factors and TMD [7,8]. The relationship is considered bidirectional: psychological distress may enhance pain perception through central sensitization and dysregulation of descending inhibitory pathways, while chronic pain may increase emotional burden. Increased psychological stress, depression, anxiety, and chronic emotional tension are associated with increased muscle tension, especially in the facial muscles, neck, and shoulder girdle [9,10]. In the case of the masticatory muscles, such as the masseter and temporalis, chronic tension can result in overload, pain, and even inflammation.

Psychological disorders such as depression and anxiety have been shown to be associated with increased pain intensity and reduced treatment effectiveness in patients with chronic pain conditions, including TMD [11]. Individuals experiencing psychological distress may also exhibit increased masticatory muscle activity and more frequent parafunctional tooth contacts. In addition, anxiety-related behaviors such as teeth clenching and other oral parafunctions may further contribute to functional overload of the masticatory system [11].

Another psychological factor relevant to TMD is somatization, which manifests as physical symptoms originating from psychological distress, including headaches, nausea, or gastrointestinal complaints. Somatization frequently co-occurs with TMD and affects approximately 14–17% of the general population [12]. Some etiological factors may be common to both somatization and TMD, with high levels of emotional stress that warrant particular attention [13].

Anxiety, depression and prolonged stress may cause unconscious parafunctional habits, with bruxism being the most common [5,14]. Bruxism is characterized by repetitive and sustained contractions of the masticatory muscles, particularly the masseter and temporalis, leading to increased muscle activity. Prolonged or repetitive jaw-muscle activity may result in muscle fatigue, mechanical overload, and, over time, adaptive structural changes such as increased muscle thickness. Therefore, bruxism represents a potential functional link between psychological factors and alterations in masticatory muscle morphology. Bruxism also puts considerable strain on the temporomandibular joint, which may result in joint displacement, clicking or popping sounds, limited mobility, and pain radiating to nearby structures such as the ear or temple [5,14].

It has been shown that serotonin and norepinephrine play a role in inhibiting central nociceptive input, which may explain why disruptions in the regulation of these neurotransmitters, underlying depression, exacerbate pain symptoms in these patients [15]. This may also explain the pain-relieving effect of antidepressant medications [16]. On the other hand, selective serotonin reuptake inhibitors (SSRI) widely used in the treatment of depression and anxiety tend to cause sleep bruxism [17].

The evaluation of muscle structure can be performed using various imaging techniques, including computed tomography, magnetic resonance imaging, and ultrasound. Currently, ultrasound is a readily available, low-cost, and non-invasive method that provides accurate visualization of the structure and dimensions of the masticatory muscles [18]. Several studies have examined masticatory muscle thickness using ultrasound to understand the relationship between muscle dimensions and morphofunctional factors such as age, sex, bite force, facial type, arcade shape, and occlusion [18,19].

Although the association between psychological factors and TMD symptoms is well documented, it remains unclear whether psychological distress is associated with measurable structural changes in masticatory muscles. Only a limited number of studies have explored the relationship between psychological variables and ultrasonographically assessed muscle thickness. For example, Ahısha et al. [20] evaluated the relationship between psychological distress and masseter muscle thickness in patients with chronic neck pain, while Öçal et al. [21] analyzed the association between anxiety levels and masseter muscle thickness in patients with disc displacement. However, neither of these studies investigated patients with TMD-related myalgia or evaluated both masseter and temporalis muscles simultaneously.

Therefore, the existing literature provides limited evidence regarding whether psychological factors are associated with morphological characteristics of the masticatory muscles in patients with TMD-related myalgia.

Thus, the aim of this study was to assess the relationship between the thickness of masticatory muscles observed in ultrasound images and psychological factors among TMD patients with myalgia. The study hypothesized that TMD patients would show a stronger positive correlation between masticatory muscle thickness and psychological factors such as depression, anxiety, and somatization compared to individuals without TMD.

## 2. Materials and Methods

This study was designed as a cross-sectional study. The study was approved by the Bioethics Committee of the Medical University of Lublin (decision number KE-0254/160/2019), and all participants provided informed consent prior to participation. It involved 106 female students of the Medical University of Lublin aged 18 to 35, who volunteered to participate between April 2021 and June 2022. The selection of medical university students as the study population was intentional, as it allowed access to a relatively homogeneous group in terms of educational background, socioeconomic status, and health awareness. Additionally, this population is characterized by increased exposure to academic stress, which may influence psychological well-being and, consequently, TMD-related symptoms [6]. To ensure greater sample homogeneity and to reduce potential confounding effects associated with sex-related differences, we limited the study population to women within this age range. This decision was based on evidence indicating that both sex and age are associated with the prevalence of TMD [3,22,23], and that females generally present thinner masticatory muscles compared to males [18]. The inclusion criterion for the study group was the presence of myalgia diagnosed based on a clinical examination according to the Diagnostic Criteria for Temporomandibular Disorders (DC/TMD) [24]. Participants in the control group were selected based on the absence of symptoms of temporomandibular disorders. Exclusion criteria included systemic diseases, ongoing orthodontic treatment, malocclusions, and pregnancy. A total of 55 individuals were included in the study group, while 51 were assigned to the control group. This sample size was consistent with guidelines assuming a medium effect size and a power level of 70% [25]. A comparison of the study and control groups in terms of age and range of mouth opening is presented in Table 1.

The Diagnostic Criteria for Temporomandibular Disorders (DC/TMD) is a standardized and validated diagnostic protocol developed to ensure reliable and reproducible assessment of TMD. It consists of two axes: Axis I focuses on clinical conditions based on a structured clinical examination, while Axis II evaluates psychosocial status and pain-related disability using validated questionnaires. The DC/TMD protocol has been formally validated and officially translated into Polish, and the validated Polish version was used in the present study [24]. The diagnosis of myalgia was established according to Axis I criteria, which include a history of pain modified by jaw function during last 30 days and confirmation of familiar pain during standardized examination of the masticatory muscles. Since subclassification of myalgia according to the DC/TMD criteria (local myalgia, myofascial pain with spreading, and myofascial pain with referral) was not essential for the objectives of the present study, we elected not to differentiate between these subtypes. For the sake of methodological simplicity and clarity, the analysis was therefore limited to the general diagnosis of myalgia.

The DC/TMD examinations were performed at the Dental Clinical Centre of the Medical University in Lublin by M.L.-R. under the supervision of J.S., both of whom are licensed dentists and experienced in the clinical assessment of temporomandibular disorders. The DC/TMD protocol ensures high diagnostic validity and inter-examiner reliability and is widely used in both clinical and research settings.

In line with DC/TMD guidelines, the following questionnaires were used to determine the presence of psychological factors:

− Patient Health Questionnaire-9 (PHQ-9)—for the preliminary diagnosis and assessment of depression severity,
− Generalized Anxiety Disorder-7 (GAD-7)—to evaluate the severity of anxiety disorders.
− Patient Health Questionnaire-15 (PHQ-15)—to evaluate the severity of the patient’s nonspecific somatic symptoms.

All questionnaires in the local language version were downloaded from the website https://www.phqscreeners.com/select-screener (accessed on 4 January 2019). The PHQ-9, GAD-7, and PHQ-15 questionnaires were selected because they are validated, widely used, and recommended within the DC/TMD Axis II framework for the assessment of depression, anxiety, and somatic symptom burden. All instruments were completed by the participants during the same session. Missing data were handled in accordance with the DC/TMD guidelines, which include predefined rules for managing incomplete responses, such as calculating scores based on available items when permitted or excluding questionnaires with excessive missing data [24].

**Figure 1:**
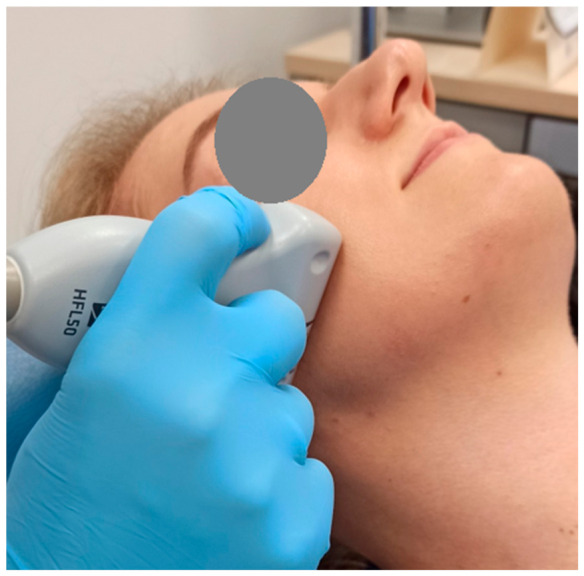
Placement of ultrasound probe during measurement of masseter muscles thickness.

**Figure 2:**
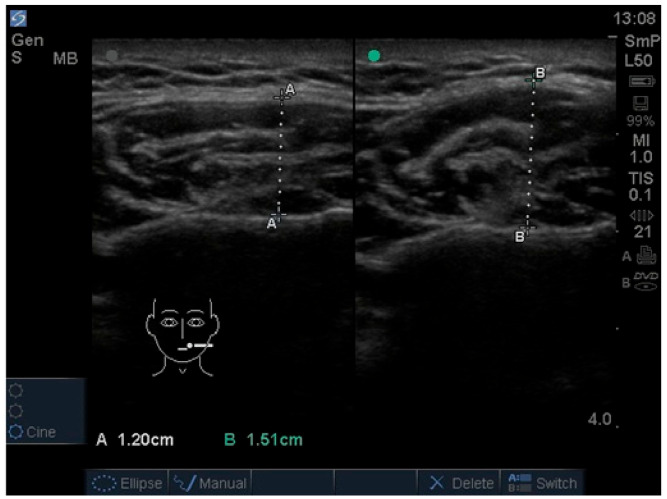
Ultrasound image of masseter muscle. Relaxed muscle on the left (measurement **A**). Contracted muscle on the right (measurement **B**).

**Figure 3:**
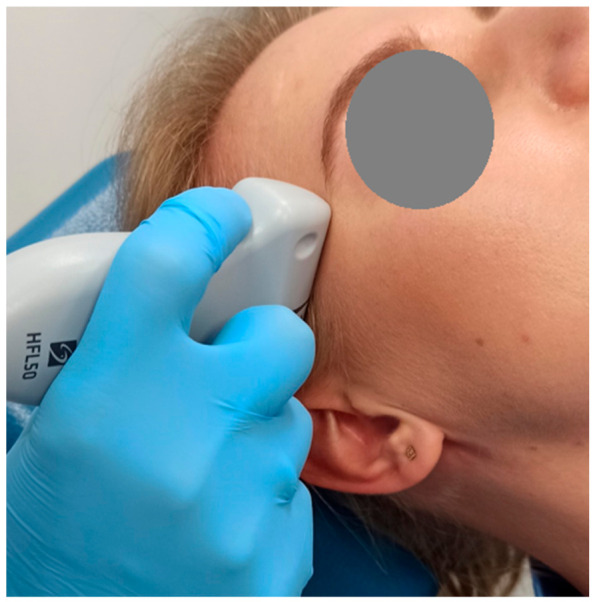
Placement of ultrasound probe during measurement of temporalis muscles thickness.

**Figure 4:**
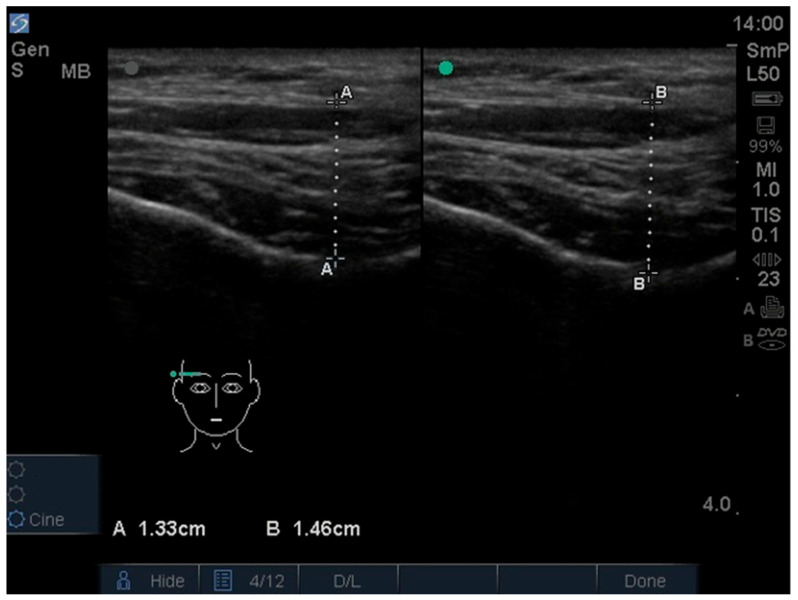
Ultrasound image of temporalis muscle. Relaxed muscle on the left (measurement **A**). Contracted muscle on the right (measurement **B**).

The thickness of the temporalis and masseter muscles was assessed using an M-Turbo ultrasound device with a linear probe, operating within a frequency range of 6 to 15 MHz and an imaging depth of up to 6 cm (SonoSite Inc., Bothell, WA, USA) [26]. All ultrasonographic measurements were performed by M.W. under the supervision of I.R.-K., a board-certified radiologist with many years of clinical experience in radiology. The masseter muscle was examined at the height of the occlusal plane, with the probe perpendicular to the ramus of the mandible and parallel to the occlusal plane (Figure 1 and Figure 2) [27]. The temporalis muscles were examined in their anterior part, with the probe placed parallel to the zygomatic arch and 1 cm above it, and perpendicular to the temporal bone, anterior to the hairline (Figure 3 and Figure 4) [28]. The selection of these measurement sites was based on a review of previous ultrasonographic studies on masticatory muscles. The adopted locations are among the most commonly used in the literature and provide high repeatability of measurements. Each muscle was measured in two conditions: during relaxation with minimal contact between antagonistic teeth, and during maximum voluntary clenching. The probe was applied with as little pressure as possible, with additional ultrasound gel used to minimize tissue compression. Muscle thickness was measured directly on the screen at the thickest visible point using the ultrasound device’s built-in measuring tool, with an accuracy of 0.1 mm. During the examination, participants were positioned in the supine position with the head comfortably supported on a headrest to ensure stabilization and standardization of measurements. The entire examination was performed twice, yielding four measurements for each muscle (two during relaxation and two during contraction).

Each participant was examined on a single day within the same facility to ensure consistency of the patient’s condition across all assessments and to minimize the risk of dropouts. The assessments were conducted in the following sequence: clinical examination, questionnaire assessment, and ultrasonographic evaluation.

For statistical analysis, the average of the two measurements was used, along with the ratio of muscle thickness during contraction to muscle thickness at rest, calculated by dividing the thickness during contraction by the thickness at rest. The error in muscle thickness measurement using ultrasound was calculated based on the difference between the first and second measurements for each muscle according to Dahlberg’s formula [29]:

$$SE=\sqrt{\frac{\sum d^{2}}{2n}}$$

where:

SE = Standard Error
d—difference between first and second measurement
n—number of examined patients

Absolute error (expressed in millimeters) reflects the magnitude of measurement variability, while relative error (expressed as a percentage) represents this variability in relation to the mean muscle thickness, allowing comparison between muscles and measurement conditions.

The results are presented in Table 2.

Descriptive statistics were used to summarize the data and included the mean, median, standard deviation, minimum, and maximum values. The normality of the variable distribution was checked using the Shapiro–Wilk test. Inferential statistical methods were applied to analyze differences and relationships between variables. The Mann–Whitney U test was used to determine differences between groups. Correlations were assessed using Spearman’s rho coefficient. The Spearman rho coefficient (ρ) ranges from −1 (perfect negative monotonic association) to +1 (perfect positive monotonic association). A correlation was considered strong for values greater than 0.5, and moderate for values between 0.3 and 0.5 [25,30]. Differences between correlations were tested using Fisher’s *z* test. The significance level was set at *p* < 0.05. Effect size was calculated according to Tomczak et al. [31].

## 3. Results

The average thickness of the temporalis muscles in the study group was 12.41 mm, while in the control group, it was 12.47 mm. For the masseter muscles, the thickness was 12.91 mm in the study group and 13.07 mm in the control group. The ratio of temporalis muscle thickness during contraction to its thickness at rest averaged 1.049 in the study group, while it was 1.054 in the control group. Similarly, the ratio for the masseter muscles was 1.091 in the study group and 1.087 in the control group. No statistically significant association was found between the occurrence of TMD and any of these muscle parameters (Table 3).

In the study group, the average score on the PHQ-9 questionnaire was 8.91, compared to 4.98 in the control group. For the GAD-7 questionnaire, the average score was 8.57 in the study group and 4.67 in the control group. For the PHQ-15, the average score was 9.57 in the study group and 5.98 in the control group. A statistically significant relationship was established between the results of each questionnaire and the occurrence of TMD (Table 4).

A statistical analysis was conducted to examine the correlation between masticatory muscle parameters and questionnaire scores, separately for both the study and control groups, followed by a comparison of these correlations. A statistically significant relationship was found between the PHQ-9 score and temporalis muscle thickness in the study group. Similarly, in the control group, a significant relationship was noted between the GAD-7 score and temporalis muscle thickness. Additionally, the differences in correlation coefficients between the study and control groups for temporalis muscle thickness and the scores on the PHQ-9 and GAD-7 questionnaires were statistically significant (*p* = 0.011 and *p* = 0.002, respectively). In the study group, higher scores on the PHQ-9 or GAD-7 questionnaires were associated with thicker temporalis muscles. In contrast, the control group exhibited the opposite trend: higher questionnaire scores corresponded to thinner temporalis muscles. Differences in other correlation coefficients did not reach statistical significance (Table 5).

## 4. Discussion

Results of our research partially confirmed the stated hypothesis. Statistically significant differences in the relationships between the study and control groups were found only for the temporalis muscle and anxiety disorders and depression. No statistically significant correlation was observed for the masseter muscles. Furthermore, somatization did not show statistically significant differences in the relationship with masticatory muscle thickness between the control and study groups.

Importantly, the presence of myalgia does not necessarily imply structural muscle hypertrophy. Myofascial pain is primarily associated with altered neuromuscular function, increased muscle activity, peripheral and central sensitization, and local ischemic or metabolic changes rather than with permanent morphological enlargement of the muscle. Therefore, the absence of significant differences in masseter muscle thickness between the study and control groups in our research should not be interpreted as contradictory to the presence of muscle-related pain. Pain may occur independently of measurable structural changes detectable by ultrasonography.

Our findings support the concept that TMD-related myalgia may reflect functional and neurophysiological alterations rather than macroscopic structural remodeling of the masticatory muscles. This distinction is clinically relevant, as it suggests that increased muscle thickness should not be considered a necessary diagnostic or pathophysiological marker of myogenous TMD.

These findings suggest that the role of psychological factors in TMD may be primarily reflected in pain perception and functional alterations rather than in measurable structural muscle changes. Psychological distress may influence muscle activity patterns, central pain modulation, and behavioral responses without necessarily leading to detectable morphological remodeling. Therefore, the absence of significant differences in muscle thickness does not diminish the clinical relevance of psychological variables in TMD.

Our study revealed a strong correlation between each of the evaluated psychological factors and TMD, aligning with the findings of the literature [11,12,13,32,33,34,35]. These results further support the growing body of evidence highlighting the significant impact of psychological factors on the development and progression of TMD.

Numerous studies have explored variations in masticatory muscle thickness, with the masseter and temporalis muscles being the most frequently examined due to their accessibility via ultrasound. Previous research has examined the thickness of these muscles in relation to bite force [36,37], muscle electrical activity [38], nocturnal bruxism [39], facial morphology [36], body structure [40], occlusal disorders [40], tooth wear [41], stabilization splint use [42], muscular dystrophy [43], temporomandibular joint osteoarthritis [44], and submucosal fibrosis [45].

The average muscle thickness largely depended on the measurement site. In studies with similar methodology, masseter thickness ranged from 9.47 to 12.74 mm at rest [27,36,41,42,45,46,47] and from 11.03 to 16.65 mm during contraction [27,36,41,45,46,47], while temporalis thickness ranged from 7.12 to 13.1 mm at rest [41,46,47] and from 8.05 to 14.4 mm during contraction [41,46,47], aligning with our study’s results. Two studies examined the ratio of masseter thickness in contraction to rest, reporting an average ratio of 1.24 to 1.42 [41,48]. One study assessed the same relationship for the temporalis muscle, with an average of 1.2 [41]. Both of these ratios are higher than the values obtained in our study, possibly due to differences in the force applied by the ultrasound probe, as our contraction measurements were similar, but there were notable differences in the resting state, where probe pressure has a greater impact. In general, the ranges of muscle thickness reported in these studies are consistent with our findings, suggesting that our measurements fall within the expected physiological variability.

Five studies explored the relationship between TMD and masticatory muscle thickness. Marcelo Palinkas et al. [39] examined the relationship between masseter and temporalis thickness and bruxism, using polysomnographic assessment as the diagnostic standard. Similarly to our study, they measured muscle thickness at rest and during contraction. Luciano Jose Pereira et al. [37] investigated the correlation between masseter and temporalis thickness and TMD symptoms using the craniomandibular index (CMI), measuring muscle thickness in both rest and contraction. Mariah Acioli Righetti et al. [44] assessed the link between the thickness of these muscles and temporomandibular joint osteoarthritis, with a diagnosis based on American College of Rheumatology guidelines. Hasan Erkan Kilinc et al. [49] explored the association between muscle thickness and myofascial pain, finding no significant differences between TMD patients and controls in terms of muscle thickness, consistent with our results.

This distinction becomes particularly relevant in the context of bruxism. Increased muscle thickness, especially of the masseter, may occur in individuals with bruxism and often presents as hypertrophy without accompanying pain. In such cases, structural adaptation may result from repetitive mechanical loading rather than from pain-related mechanisms. Our results support the concept that muscle hypertrophy and myalgia represent distinct phenomena that may coexist but are not causally dependent on one another.

The literature also discusses the relationship between psychological factors and TMD. Louis Simoen et al. [50] examined the link between anxiety, depression, and TMD-related pain, using the PHQ-9 and GAD-7 questionnaires, and found a strong correlation between depression, anxiety, and TMD pain, which aligns with our findings. Ema Vrbanović et al. [51] also found that psychological factors such as depression and anxiety correlated with increased pain in TMD patients, but they did not find this correlation when comparing TMD patients to healthy individuals, possibly due to group selection differences. The COVID-19 pandemic, which heightened psychological distress, may have affected their results.

Chengxinyue Ye et al. [52] also demonstrated strong correlations between TMD and both depression and anxiety, using DC/TMD criteria and PHQ-9 and GAD-7 questionnaires, in line with our results. Birgitta Häggman-Henrikson et al. [53] and Adrian Ujin Yap et al. [54] both showed correlations between somatization and TMD, as well as between psychological distress and TMD, matching our findings.

Among the available literature, only two studies assessed the relationship between masticatory muscles thickness and psychological factors. Ahısha et al. [20] examined the correlation between the masseter muscle thickness and depression, anxiety, and somatization using the same questionnaires (PHQ-9, GAD-7 and PHQ-15) in female patients with chronic non-specific neck pain. They demonstrated significant positive correlation between masseter thickness and scores on PHQ-9 and GAD-7 in the studied population.

Öçal et al. [21] also assessed the relationship between masseter thickness and psychological factors. They found that patients with unilateral disc displacement and higher anxiety levels had thicker masseters on the unaffected side compared to patients with normal anxiety levels. However, they reported no such relationship on the affected side.

In contrast, in our study, no significant correlation was observed between masseter muscle thickness and psychological factors. The discrepancies between our findings and those reported by Ahısha et al. may be attributed to differences in the studied populations. While their research focused on female patients with chronic non-specific neck pain, our study included individuals diagnosed with TMD-related myalgia. Furthermore, our participants were considerably younger (mean age: 23 years) compared to their cohort (mean age: 41 years). Age-related variations in muscle morphology, adaptive capacity, and chronicity of symptoms, as well as differences in the underlying pain condition, may have influenced the observed associations. Similarly, differences between our results and those of Öçal et al. may be explained by variations in study design and population characteristics. We examined exclusively female patients with myalgia, whereas Öçal et al. included both male and female patients diagnosed with disc displacement. These clinical and demographic differences may account for the divergent findings. Notably, neither of these studies evaluated temporalis muscle thickness, which further limits direct methodological comparability with our investigation. Taken together, these findings suggest that the relationship between psychological factors and masticatory muscle morphology may be context-dependent and influenced by age, type of TMD, and study design.

The present study has several strengths. First, it combined standardized clinical diagnostics based on the DC/TMD protocol with validated psychological assessment tools (PHQ-9, GAD-7, and PHQ-15) and objective ultrasonographic measurements of masticatory muscle thickness. Second, all assessments were performed on the same day and within the same facility, which minimized temporal variability and ensured consistency of the participants’ clinical condition across examinations. Third, ultrasonographic measurements were conducted under standardized conditions and supervised by an experienced radiologist, enhancing methodological reliability. Finally, the study addressed a relatively underexplored research question by examining the association between psychological factors and muscle thickness in TMD-related myalgia.

Several limitations should be acknowledged. First, psychological factors were assessed exclusively using self-report questionnaires without formal psychiatric evaluation, which may limit diagnostic precision. Second, we did not control for potential confounders such as facial morphology or body mass index, both of which may influence masticatory muscle thickness. Third, the study population consisted exclusively of young female dental students, which enhances internal homogeneity but limits the generalizability of the findings to broader and more diverse populations. Additionally, the cross-sectional design precludes causal inference regarding the relationships observed.

## 5. Conclusions

− Individuals with myalgia did not show increased muscle thickness compared to healthy individuals.
− Individuals with myalgia exhibited more symptoms of depression, anxiety, and somatisation.
− A significant association between depression and anxiety scores and temporalis muscle thickness was identified; however, the direction of this association differed between groups. In the myalgia group, higher PHQ-9 and GAD-7 scores were associated with greater temporalis thickness, whereas in the control group, higher scores were associated with lower temporalis thickness.
− No significant associations were found between psychological factors and masseter muscle thickness.

## Figures and Tables

**Table 1:** Comparison between study and control group in terms of age and range of opening.

	Study Group (*n* = 55)	SD	Control Group (*n* = 51)	SD	U	*p*
Age [years]	23.49	2.31	23.35	2.50	1274.00	0.41
Pain-free opening [mm]	46.69	9.03	48.86	5.66	1269.50	0.40
Maximum unassisted opening [mm]	50.20	5.60	49.18	5.85	1208.00	0.22
Maximum assisted opening [mm]	53.38	5.64	51.82	5.76	1150.50	0.11

SD: standard deviation; U: U—Mann–Whitney U test statistic; *p*—significance level.

**Table 2:** Measurement error of muscle thickness using ultrasound examination.

	Measurement Error of Realxed Muscle		Measurement Error of Contracted Muscle	
	Absolute [mm]	Relative [%]	Absolute [mm]	Relative [%]
**left temporalis muscle**	0.88	7.15	0.86	6.61
**right temporalis muscle**	1.01	8.09	1.04	7.91
**left masseter muscle**	0.62	4.76	0.55	3.96
**right masseter muscle**	0.63	4.85	0.50	3.52

**Table 3:** Comparison of masticatory muscle thickness measured by ultrasound.

	Study Group (*n* = 55)	SD	Control Group (*n* = 51)	SD	U	*p*	ES
**TM R [mm]**	12.47	1.70	12.40	1.24	1385.50	0.92	0.05
**MM R [mm]**	13.09	2.02	12.89	1.49	1386.00	0.92	0.11
**TM C/R**	1.05	0.03	1.05	0.02	1276.50	0.43	0.20
**MM C/R**	1.09	0.04	1.09	0.04	1310.00	0.56	0.08

SD: standard deviation; U: U—Mann–Whitney U test statistic; *p*—significance level; ES—effect size; TM R: average resting temporalis muscle thickness; MM R: average resting masseter muscle thickness; TM C/R: average ratio of temporalis muscle thickness during contraction to its thickness at rest; MM C/R average ratio of masseter muscle thickness during contraction to its thickness at rest.

**Table 4:** Comparison of questionnaire results for assessing psychological factors based on DC/TMD.

	Study Group (*n* = 55)	SD	Control Group (*n* = 51)	SD	U	*p*	ES
**PHQ 9**	8.82	5.02	5.00	3.41	760.50	<0.001	0.88
**GAD 7**	8.53	4.48	4.65	3.58	674.50	<0.001	0.95
**PHQ 15**	9.47	4.07	6.02	3.89	724.50	<0.001	0.87

SD: standard deviation; U: U—Mann–Whitney U test statistic; *p*—significance level; ES—effect size; PHQ 9: average score of patient health questionnaire 9; GAD 7: average score of general anxiety disorders; PHQ 15: average score of patients health questionnaire 15; DC/TMD: Diagnostic Criteria/Temporomandibular Disorders.

**Table 5:** Correlations between questionnaire results assessing psychological factors and masticatory muscles thickness in ultrasound examination.

		Study Group (*n* = 55)		Control Group (*n* = 51)		*p* (Fisher *z*-Test)	ES
		*r*	*p*	*r*	*p*		
**TM R**	PHQ 9	0.32	0.020 *	−0.13	0.359	0.011 *	0.46
	GAD 7	0.25	0.066	−0.30	0.030 *	0.002 *	0.57
	PHQ 15	0.18	0.183	−0.13	0.365	0.058	0.31
**MM R**	PHQ 9	0.20	0.139	0.15	0.291	0.389	0.05
	GAD 7	0.14	0.299	−0.12	0.393	0.091	0.26
	PHQ 15	−0.08	0.551	−0.16	0.251	0.344	0.08
**TM C/R**	PHQ 9	0.08	0.565	0.25	0.072	0.188	0.18
	GAD 7	0.02	0.874	0.10	0.459	0.339	0.08
	PHQ 15	0.12	0.380	0.07	0.610	0.402	0.05
**MM C/R**	PHQ 9	−0.10	0.476	0.17	0.228	0.088	0.27
	GAD 7	−0.14	0.317	0.14	0.324	0.081	0.28
	PHQ 15	−0.11	0.437	0.07	0.618	0.185	0.18

*r*: Spearman’s rank correlation coefficient; *p*—significance level; ES—effect size; *—indicates statistical significance at the p < 0.05 level; TM R: resting temporalis muscle thickness; MM R: resting masseter muscle thickness; TM C/R: ratio of temporalis muscle thickness during contraction to its thickness at rest; MM C/R ratio of masseter muscle thickness during contraction to its thickness at rest; PHQ 9: health questionnaire 9; GAD 7: general anxiety disorders; PHQ 15: health questionnaire 15.

## Data Availability

Data are available upon reasonable request from the corresponding author.

## Abbreviations

The following abbreviations are used in this manuscript:

TMD	Temporomandibular disorders
DC/TMD	Diagnostic criteria for temporomandibular disorders
PHQ-9	Patient health questionnaire 9-item depression scale
GAD-7	Generalized anxiety disorder 7-item anxiety scale
PHQ-15	Patient health questionnaire 15-item somatic scale
